# The Hidden Cost of Asthma Control: Esophageal Candidiasis Induced by Inhaled Corticosteroids

**DOI:** 10.1155/carm/5532765

**Published:** 2025-10-24

**Authors:** Amal Miqdadi, Fahd Ghalim, Mohamed Reda Cherkaoui Jaouad, Abdennaceur El Idrissi Lamghari, Abderrahmane Al Bouzidi, Mohammed Herrag

**Affiliations:** Faculty of Medicine, Cheikh Khalifa International University Hospital, Mohammed VI University of Sciences and Health, Casablanca, Morocco

**Keywords:** asthma, complication, esophageal candidiasis, inhaled corticosteroids, misuse

## Abstract

The misuse of inhaled forms of corticosteroids may expose one to oesophageal candidiasis. In our case, the patient used to rinse his mouth with water and to swallow it after each inhalation of FP. Considering their lipophilic characteristic, the laboratories should manufacture a new efficacious hydrophilic inhaled corticosteroid.

## 1. Introduction

Inhaled corticosteroids (ICS) have long been considered the cornerstone of asthma management. According to the recent Global Initiative for Asthma (GINA) recommendations, they can be used on an as-needed basis in combination with bronchodilators, in addition to their established role as reliever therapy during exacerbations [1]. Although generally well tolerated, ICS may cause local side effects such as dry mouth, hoarseness, oropharyngeal, and laryngeal candidiasis [2]. Esophageal candidiasis (EC) is a much less commonly reported complication, often overlooked in clinical practice.

## 2. Case Report

A 67-year-old man was admitted for a severe asthma exacerbation. Two days earlier, he developed chest tightness, a productive cough with greenish sputum, oppression, and wheezing, which did not resolve despite his regular asthma treatment with inhaled fluticasone propionate (FP, 1000 μg/day) and salmeterol, a long-acting β2-agonist (LABA, 100 μg/day). On questioning, he had a long history of asthma since the age of 16, but during the last 3 years he self-managed his disease without medical supervision, using FP/salmeterol both as maintenance and as reliever therapy, averaging two canisters per month. After each inhalation, he rinsed his mouth with water and swallowed it. He had not received systemic corticosteroids during the previous 3 years.

Two years earlier, in addition to his respiratory symptoms, he developed intermittent hoarseness treated with over-the-counter medication, which progressively worsened and became persistent 6 months ago. He also reported recent epigastralgia with heartburn. He denied dysphagia, odynophagia, metallic taste, nausea, vomiting, weight loss, or changes in bowel habits.

**Figure 1:**
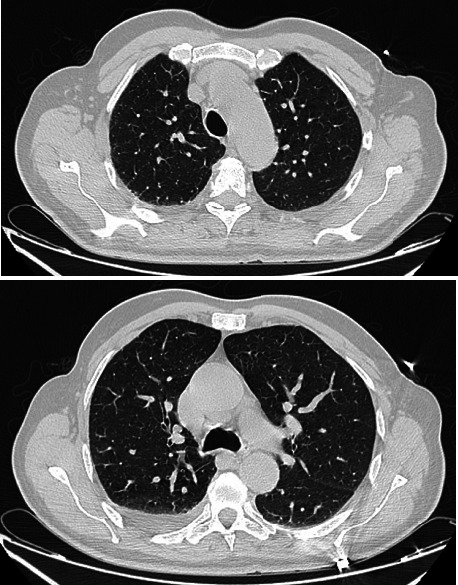
Axial CT-scan views showing bronchial wall thickening associated to a small right pleural effusion.

On examination, diffuse wheezing was present, but no perleche or cheilitis was noted. The remainder of the physical exam was unremarkable. Chest CT revealed bronchial wall thickening with a small right pleural effusion (Figure 1). Laboratory investigations showed leukocytosis (21,900/mm^3^, predominantly neutrophils), lymphopenia (920 elements/mm^3^), 530 elements/mm^3^ of eosinophils, elevated CRP (123 mg/L), and negative results for infectious workup including COVID-19 PCR, acid-fast bacilli smear, multiplex respiratory panel, HIV, and hepatitis B and C serologies.

**Figure 2:**
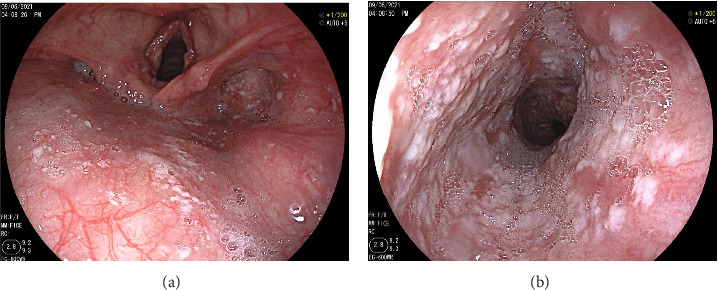
Images of disseminated oropharyngeal and esophageal candidiasis seen on oesogastroscopy exploration. (a) Whitish deposits in the oropharynx. (b) An appearance of disseminated esophageal candidiasis.

**Figure 3:**
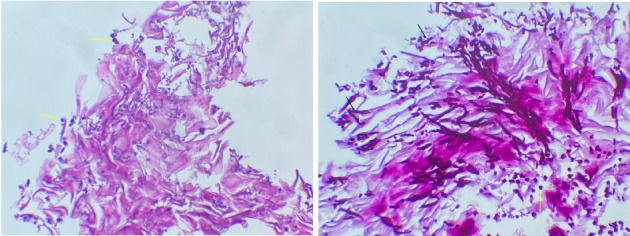
Histopathological analysis showing parakeratotic leukokeratosis with numerous *Candida* spores (yellow arrow) and hyphae (black arrow).

Despite broad-spectrum antibiotics (third-generation cephalosporins and fluoroquinolones) and a proton-pump inhibitor (80 mg/day for 3 days), the patient's asthma exacerbation improved within 24 h, but his gastric symptoms persisted. Consequently, upper GI endoscopy was performed and revealed whitish plaques in the oropharynx (Figure 2(a)) and disseminated EC (Figure 2(b)). Notably, vocal cord changes were also observed, which may account for his persistent hoarseness. Histopathology confirmed candidal esophagitis, showing parakeratotic leukokeratosis with numerous *Candida* spores and hyphae (Figure 3).

The patient was treated with fluconazole (800 mg on day 1, then 400 mg/day for 10 days, followed by 150 mg/day for 3 weeks). His gastrointestinal complaints and hoarseness progressively resolved over the following 3 weeks.

## 3. Discussion

ICS remain the cornerstone of asthma management, improving symptoms, pulmonary function, and survival [3]. However, their prolonged or inappropriate use can lead to local side effects such as oropharyngeal and laryngeal candidiasis [2]. EC is a far less commonly reported complication, often overlooked in daily practice.

Although the prevalence of EC among patients receiving high-dose FP may reach 36% on endoscopic evaluation [4], these cases are usually asymptomatic. Therefore, symptomatic EC in immunocompetent patients is rare, which underscores the relevance of our case.

The risk of EC is linked to the pharmacological properties of FP. This drug has a strong affinity for type II glucocorticoid receptors [5], high lipophilicity, and potent anti-inflammatory activity [6, 7]. These features allow efficacy at low concentrations but also promote prolonged mucosal retention. Up to 70% of the inhaled dose deposits in the oropharynx and is subsequently swallowed [8], exposing the esophagus to steroids and predisposing it to *Candida* overgrowth. In our patient, the risk was further increased by incorrect mouth rinsing technique, since he swallowed water after each inhalation rather than spitting it out.

The clinical spectrum of EC is variable. Classical symptoms include dysphagia, odynophagia, retrosternal pain, or metallic taste, but many patients remain asymptomatic [2, 4]. Our patient presented with hoarseness, epigastralgia, and heartburn, symptoms initially attributed to gastroesophageal reflux disease (GERD). Endoscopy confirmed EC with associated vocal cord changes, explaining his long-standing hoarseness. This highlights the need for laryngeal inspection in ICS users with persistent voice changes.

GERD is highly prevalent in asthma patients and may overlap with candidiasis, making differentiation clinically challenging. Our case illustrates that persistent reflux-like symptoms despite proton-pump inhibitor therapy should raise suspicion for EC, especially in high-dose ICS users.

Management relies on antifungal therapy, with fluconazole being highly effective in immunocompetent individuals [2, 4]. In our patient, treatment led to complete resolution of symptoms and improvement in asthma control. Preventive measures are crucial: prescribing the lowest effective ICS dose, using valved holding chambers to enhance pulmonary deposition [9], and educating patients to rinse and spit after each inhalation. Given the lipophilic nature of most ICS, rinsing with water may be insufficient. Future research should focus on hydrophilic steroid formulations or adjunctive rinsing solutions to reduce mucosal exposure.

This case describes a rare but clinically significant case of symptomatic EC in an immunocompetent asthma patient misusing high-dose FP. Clinicians should remain vigilant for this complication, as early recognition and treatment can prevent prolonged morbidity and improve asthma outcomes.

## 4. Conclusion

EC should be considered as a potential complication of prolonged or inappropriate ICS use, even in immunocompetent patients. Clear patient education on correct inhaler technique and proper mouth rinsing is essential. Clinicians should remain vigilant for atypical symptoms such as hoarseness or refractory reflux, and timely endoscopy should be performed when indicated. Future development of safer, more hydrophilic corticosteroid formulations may help reduce this risk.

## Nomenclature

GINA: Global initiative for asthma
FP: Fluticasone propionate
CRP: C-reactive protein
PCR: Polymerase chain reaction
